# Clinical Effects of Standard and Individualized Dialysate Sodium in Patients on Maintenance Hemodialysis

**DOI:** 10.3889/oamjms.2016.056

**Published:** 2016-05-10

**Authors:** Natasa Eftimovska–Otovic, Olivera Stojceva-Taneva, Risto Grozdanovski, Saso Stojcev

**Affiliations:** 1 *Specialized Hospital for Nephrology and Dialysis “Diamed”, Skopje, Republic of Macedonia*; 2 *University Clinic for Nephrology, Medical Faculty, Ss Cyril and Methodius University of Skopje, Skopje, Republic of Macedonia*; 3 *General City Hospital “8th September”, Skopje, Republic of Macedonia*

**Keywords:** blood pressure, thirst, dialysate sodium, hemodialysis

## Abstract

**BACKGROUND:**

The degree to which the dialysate prescription and, in particular, the dialysate sodium concentration influences blood pressure and interdialytic weight gain (IDWG) via changes in sodium flux, plasma volume or the other parameters is not well understood. The aim of the study was to investigate whether dialysis patients will have some beneficial effects of dialysate sodium set up according to serum sodium or sodium modeling.

**MATERIAL AND METHODS:**

Ninety-two nondiabetic subjects (52 men and 40 women) performed 12 consecutive hemodialysis (HD) sessions (4 weeks) with dialysate sodium concentration set up on 138 mmol/L (standard sodium – first phase), followed by 24 sessions (second phase) wherein dialysate sodium was set up according to individualized sodium. Variables of interest were: systolic, diastolic and mean blood pressure, pulse, IDWG, thirst score – (Xerostomia Inventory (XI) and Dialysis Thirst Inventory (DTI)) and side effects (occurrence of hypotension and muscle cramps). After the first phase, the subjects were divided into 3 groups: normotensive (N=76), hypertensive (N= 11) and hypotensive (N=5) based on the average pre-HD systolic BP during the whole period of the first phase.

**RESULTS:**

Sodium individualization resulted in significantly lower blood pressure (133.61 ± 11.88 versus 153.60 ± 14.26 mmHg; p=0.000) and IDWG (2.21 ± 0.93 versus 1.87 ± 0.92 kg; p=0.018) in hypertensive patients, whereas normotensive patients showed only significant decrease in IDWG (2.21 ± 0.72 versus 2.06 ± 0.65, p=0,004). Sodium profiling in hypotensive patients significantly increased IDWG (2.45 vs. 2.74, p= 0,006), and had no impact on blood pressure. Thirst score was significantly lower in normotensive patients with individualized-sodium HD and showed no change in the other two groups. During the second phase, hypotension occurred in only 1 case and muscle cramps in 10 normotensive patients.

**CONCLUSION:**

Individualized sodium resulted in clinical benefits in normotensive and hypertensive patients.

## Introduction

Prescription of dialysate sodium for patients on maintenance hemodialysis remains still unclear and not enough investigated issue. During the first years when dialysis was introduced as a renal replacement therapy for patients with end-stage renal failure, dialysate sodium prescription was 126.5 mmol/l. Before introduction of volumetric controlled ultrafiltration, sodium was removed primarily, slowly and most predictably by diffusion. With the development of high flux dialysis membranes, dialysate osmolality asserted a faster and more dramatic effect on serum osmolality. Hypotonic dialysate rapidly drops serum osmolality that leads to net fluid shift out of the vascular space, causing significant intradialytic symptoms. Furthermore, the duration of dialysis sessions was shortened as clearance of urea was improved, requiring an accelerated rate of ultrafiltration. To counter symptoms of hypo-osmolarity and rapid ultrafiltration, dialysate sodium concentration was increased to level of 140 mmol/L and higher just to maintain hemodynamic stability during dialysis and to avoid side effects of dialysis – disequilibrium. This was followed by a loss of control of extracellular volume (ECV) and blood pressure (BP) [1]. This led a lot of studies to investigate on which level the dialysate sodium should be set up. Current hemodialysis (HD) practices adopt a standard dialysate sodium prescription that is typically higher than the plasma sodium concentration of most patients. However, hypertonic dialysate sodium prescriptions, including sodium modeling, predispose to positive sodium balance and lead to higher BP and increased interdialytic weight gain [2]. Predialysis plasma sodium concentration is constant in HD patients, and these patients seem to have an individual osmolar set point with a small variances of 1-2% and this is the value on which dialysate sodium should be prescribed to eliminate the interdialytic accumulated sodium mainly by convection [3]. On the other hand, lowering or individualizing dialysate sodium aims to reduce thirst, IDWG and BP in non-hypotensive prone patients [4]. In hypotensive-prone patients, dialysate sodium modeling is very ofen used (start of HD with higher dialysate sodium and slowly lowering during the session to standrad sodium, mostly to 138 mmol/L) to keep hemodynamic stability. In approximately 10%–15% of patients, instead of decreasing, BP paradoxically increases during dialysis. These patients have intradialytic hypertension [5]. The degree to which the dialysate prescription and, in particular, the dialysate sodium concentration influences blood pressure and IDWG via changes in sodium flux, plasma volume or the other parameters is not well understood. The aim of the study was to investigate whether dialysis patients will have some beneficial effects of dialysate sodium set up according to serum sodium or sodium modeling.

## Materials and Methods

The study was carried out in a single dialysis center treating 109 patients with maintenance hemodialysis. It was performed in two different phases, with each subject used as own control. Dry weight, dialysis prescription and medications were not modified during the study, except for dialysate sodium concentration. Blood flow was in general 250 ml/min, and increased in some patients up to 290 ml/min, and dialysate flow was 500 ml/min, up to 550 ml/min in some patients. Out of 109 treated in our center, the study included 92 non-diabetic subjects on high flux bicarbonate dialysis, 3 times weekly and residual diuresis below 300 ml/day. Before the start of the study, the average pre-HD plasma sodium concentration was calculated (mean value of 12 monthly measurements). During the first phase, the patients underwent 12 consecutive HD sessions (4 weeks) with dialysate sodium concentration set up on 138 mmol/L (which accounts for a standard sodium concentration in our center). During the second phase, the patients underwent 24 HD sessions (8 weeks) wherein dialysate sodium was set to the mean value of the pre – HD plasma sodium concentration of each individual patient (individualized sodium). Patients were not aware of the modification in the dialysate sodium concentration.

Pre-, intra-and post-HD blood pressure were measured using Omron M6 comfort device. After the first phase, the subjects were divided into 3 groups: normotensive (N = 76), hypertensive (N = 11) and hypotensive (N = 5) based on the average pre-HD systolic BP during the whole period of the first phase. Hypertensive patients were defined as pre-HD systolic BP >/= 140 mmHg or an increase of more than 10 mmHg during or at the end of the session, while hypotensive patients were defined as pre-HD systolic BP </=90 mmHg or having a drop in BP of more than 10 mmHg during or at the end of the session [5]. According to the NKF k-DOQI guidelines, predialysis and postdialysis blood pressure goals should be <140/90 mmHg and <130/80 mmHg, respectively [6]. After the first phase, hypotensive-prone patients underwent dialysis with sodium modeling (145-138 mmol/L) and the other two groups underwent dialysis with individualized sodium. Variables of interest were: systolic, diastolic and mean blood pressure, pulse, IDWG, thirst score and side effects (episodes of hypotension and muscle cramps). Interdialytic fluid accumulation was derived from the difference in weight before next hemodialysis and weight at the end of the previous hemodialysis. Mean blood pressure was calculated as the sum of the systolic plus doubled diastolic pressure, divided by three. Thirst was assessed using two different scales: Xerostomia Inventory (XI) and Dialysis Thirst Inventory (DTI) [7].

The sodium was measured with direct ion selective method. This method measures non-complexed, free sodium concentration in plasma water, which represents those sodium molecules available for diffusion. If the patient was prescribed sodium modeling, we calculated the sodium gradient as the difference between the dialysate sodium averaged concentration and the pre-HD plasma sodium in the previous 12 months.

The adequacy of dialysis (spKt/V) was estimated by the Daugirdas equation [8].

Statistical analysis was performed using the statistical package SPSS Statistics 17. The results were expressed as mean (± SD). We used paired Student t-test to compare continuous variables between each study phase (the parameters of the total patient group were compared between the first and the second phase) and unpaired Student t-test was used to compare hypertensive with normostensive subjects. Pearson correlation coeficient was used to study relationship between different continuous variables. P-values <0.05 were considered statistically significant.

## Results

Ninety-two non-diabetic patients, 52 men and 40 women with dialysis vintage 78.91 ± 67.52 months were analyzed. There were no statistical significant differences in SBP, DBP, MAP and pulse for all the subjects when compared with standard sodium dialysate and individualized sodium dialyzate. There was only significant decrease in pulse, IDWG, XI and DTI score in the inidividualized sodium dialysate group (Table 1).

**Table 1:** Comparison of variables between standard-NaHD and individualized-Na HD

Variables	Standard sodium	Individualized sodium	p-value
SBP (mmHg)	124.99 ± 19.42	123.26 ± 15.77	0.128

DBP (mmHg)	74.14 ± 10.87	73.20 ± 9.72	0.095

MAP (mmHg)	87.67 ± 10.65	87.50 ± 11.08	0.759

Pulse (beats/min)	78.54 ± 20.54	73.18 ± 11.58	0.000*

IDWG (kg)	2.22 ± 0.73	2.08 ± 0.70	0.001*

Xerostomia Inventory score	17.77 ± 7.13	15.02 ± 5.59	0.000*

Dialysis Thirst Inventory score	12.70 ± 4.96	10.88 ± 4.28	0.000*

However, when the patients were categorized into three goups, patients with hypertension, hypotension-prone patients and normotensive patients, it became apparent that hypertensive patients hada significant reduction in SBP (133.61 ± 11.88 versus 153.60 ± 14.26 mm Hg; p = 0.000) during the individualized-sodium dialysis compared to standard-dialysate sodium, DBP (78.61 ± 4.73 versus 87.85 ± 6.08 mmHg; p = 0.000) and MAP (96.94 ± 5.95 versus 124.21 ± 23.80 mmHg; p = 0.008), whereas normotensive patients had a net, statistically not significant, increase in SBP, DBP and MAP. Statistical significant decrease in pulse was observed in normotensive patients, but not in hypertensive ones. During dialysis performed with standard-dialysate sodium, normotensive and hypertensive patients had similar IDWG (2.21 ± 0.72 kg and 2.21 ± 0.93 kg, respectively) which significantly decreased duringdialysis using individualized-sodium dialysate in both groups (2.06 ± 0.65 kg; p = 0.004 and 1.87 ± 0.92 kg; p = 0.018, respectively). Assessment of thirst showed statistical significant decrease in normotensive patients when standard sodium dialysis was compared to individualized-sodium dialysis (XI score 17.94 ± 6.83 versus 15.00 ± 5.60; p=0.000 and DTI score 12.60 ± 4.71 versus 10.53 ± 4.08; p= 0.000), whereas this difference was not stiatistically significant in hypertensive ones (XI score 18.00 ± 10.19 versus 13.45 ± 5.59; p = 0.817 and DTI score 11.90 ± 5.88 versus 10.27 ± 3.49; P = 0.118) (Table 2).

**Table 2:** Comparison of variables between standard-Na HD and individualized-Na HD in the three groups of patients (normotensive, hypertensive and hypotensive-prone patients)

Variables	Normotensive N=76		Hypertensive N=11		Hypotensive N=5	
Age	60.46±13.15		58.72±7.41		60.50±4.41	

sNa (mmol/L)	136.77±1.47^1^		136.36±0.24^1^		136.66±1.50	

dNa	Standard Na	Individualized Na	Standard Na	Individualized Na	Standard Na	Profiling 145-138

Sodium gradient (mmol/L)	1.21±1.49	//	1.63±0.80	//	//	//

SBP	123.46±13.86	123.92±13.51	153.60±14.26	133.61±11.88^1^	86.94±5.63	89.63±5.67

DBP	73.55±8.89	73.61±9.16	87.85 ±6.08	78.61±4.73^1^	54.05±2.32	55.02±2.07

MAP	90.18±9.53	90.38±9.68	124.21±23.80	96.94±5.95^3^	67.81±5.30	68.88±4.70

Pulse	72.79±8.75	70.04±7.42^1^	74.74±6.25	72.91±6.15	76.79±3.55	74.05±2.77

IDWG	2.21±0.72	2.06±0.65^2^	2.21±0.93	1.87±0.92^4^	2.45±0.17	2.74±0.19^5^

XI score	17.94±6.83	15.00±5.60^1^	18.00 ±10.19	13.45±5.59	17.33±3.72	19.00±4.14

DTI score	12.60±4.71	10.53±-4.08^1^	11.90±5.88	10.27±3.49	16.00±5.89	17.00±4.00

Sp Kt/V	1.49±0.27	1.50±0.24	1.42± 0.30	1.43±0.19	1.53±0.16	1.58±0.24

1 P = 0.000;

2 p = 0.004;

3 p = 0.008;

4 p =0.018;

5 p = 0.006.

Plasma sodium concentration in all three groups of patients was close to 136 mmol/l, with positive sodium gradient in normotensive and hypertensive patients during the first phase. Hypertensive patients had higher positive sodium gradient in comparison to normotensive patients. In the individualized phase of the study, there was no sodium gradient.

When the mean values of the variables in the individualized-Na HD were compared between the normotensive and hypertensive group of patients, statistically significant differences were observed in SBP, DBP, MAP, pusle, IDWG and sodium gradient (Table 3).

**Table 3:** Comparison of the mean values of variables between the normostensive and hypertensive group of patients

Variables	Normotensive patients	Hypertensive patients	p-value
SBP (mmHg)	123.92 ± 13.51	133.61 ± 11.88	0.001

DBP(mmHg)	73.61 ± 9.16	78.61 ± 4.73	0.001

MAP(mmHg)	90.38 ± 9.68	96.94 ± 5.95	0.001

Pulse (beats/min)	70.04 ± 7.42	72.91 ± 6.15	0.857

IDWG (kg)	2.06 ± 0.65	1.87 ± 0.92	0.001

Xerostomia Inventory score	15.00 ± 5.60	13.45 ± 5.59	0.841

Dialysis Thirst Inventory score	10.53 ± 4.08	10.27 ± 3.49	0.695

Sodium gradient (mmol/L)	1.21 ± 1.49	1.62 ± 0.80	0.000

The correlation between IDWG and the sodium gradient between dialysate-sodium and plasma-sodium concentration in the standard-sodium dialysis phase of the study showed statistical significance (r = 0.252; p = 0.019). But, there was no significant correlation between the sodium gradient and blood pressure in patients as a whole group, as well as in hypertensive patients only. During the individualized-sodium dialysis phase, we observed only 1 hypotensive occurrence and 3 appearances of muscle cramps in the normotensive group, whereas all the other patients remained asymptomatic.

The hypotensive group of patients underwent dialysis with sodium modeling of 145-138 mmol/L. But, nevertheless, we still observed an increase in SBP when we compared it to standard-sodium dialysis (89.63 ± 5.67 versus 86.94 ± 5.63 mm Hg; p = 0.352), DBP (55.02 ± 2.07 versus 54.05 ± 2.32 mm Hg; p = 0.623) and MAP (68.88 ± 4.70 versus 67.81 ± 5.30 mm Hg; p = 0.859) although statistically not significant. There was a statistically significant increase in IDWG compared to standard-dialysate sodium (2.74 ± 0.19 kg versus 2.45 ± 0.17 kg; p = 0.006). The thirst score didn’t show statistically significant differencewhen compared to standard- sodium dialysis (XI 19.00 ± 4.14 versus 17.33 ± 3.72; p = 0.459 and DTI 17.00 ± 4.00 versus 16.00 ± 5.89; p = 0.141).

## Discussion

Our study analyzed the short – term consequences (BP, IDWG and subjective feeling of thirst) of an individualized-sodium and sodium-modeling prescription dialysis in non-diabetic HD patients. The short-term duration of the study allowed other important parameters to remain unchanged, such as dry weight and antihypertensive medications. The most prescribed antihypertensive drugs were: angiotensin-converting enzyme inhibitors, angiotensin receptor blockers and calcium channel blockers. The main findings in our study were reduction in IDWG and improvement in predialysis BP in hypertensive patients.

When the patients were analyzed as a whole group, we didn’t find significant differences in BP, IDWG and thirst score when standard-Na HD was compared to individualized-Na HD. The same conclusion is reported by De Paulaet all [9]. In our study, the sodium gradient was significantly higher in hypertensive compared to normotensive patients, suggesting lower values of pre-HD plasma sodium concentration in patients with poorly controlled BP and higher sodium overload during the dialysis, causing thirst and volume overload. Bylinear regression analyses, Keen and Gotch and Mendoza et all. showed a statistically significant association between the magnitude of the Na+ gradient and interdialytic weight gain and blood pressurein smaller samples of HD patients [10, 11]. But, Heckinget all, reported that higher dialysate-Na prescriptions are associated with increased IDWG, but not with a higher risk for hospitalization or death. Instead, patients dialyzed with higher dialysate-Na concentrations had a significantly lower risk for hospitalization and, in facilities where all or almost all patients used the same dialysate-Na, a significantly lower risk for death [12]. Individualizing the dialysate-sodium is a simple complementary strategy to restrict sodium in HD that may help reduce IDWG in some patients [13, 14]. In our study, we did not find a direct correlation between the sodium gradient and BP, which was confirmed by other investigators, too [11]. This might be a result of the use of antihypertensive agents, which may mask such correlation.

After categorizing the patients into three groups, it appeared that hypertensive patients had statistically significant decrease in BP compared to normotensive patients. The drop of BP appeared very soon, after changing thedialysate sodium, which overcame the “lag period” reported in the world literature [15]. This drop was probably a result of a better sodium balance and lower peripheral vascular resistance. We, also, found a significant decrease in IDWG in normotensive and hypertensive patients during the individualized-Na HD, suggesting no sodium overload during HD, which otherwise, forces the patient to drink more in order to bring own osmolarity back to its “set point”. This was also confirmedin hypotensive-prone patients who were dialysed with sodium modeling. Even though ending the dialysis session with a dialysate-Na of 138 mmol/L, these patients most probably had sodium overload during their HD, which led them to interdialytic fluid intake (IDWG 2.74 ± 0.19 kg in profiling Na vs. 2.45 ± 0.17 kg in standard-Na; p = 0.006). Sodium overload during sodium modeling was reported by Oliver and Lam, too [16, 17]. Hypotensive patients in our study did not have increased thirst, probably as a result of their regularsalt and fluid intakeaiming to increase their BP at home.

Individualization of dialysate-Na was very well tolerated by patients, probably as a result of the lower IDWG and lower UF rate, with almost no adverse events (one case of hypotension and few cases with muscle cramps). But, on the other hand, aiming to reach eunatremia may increase the risk of intradialytic hypotension. Indeed, two studies reported a reduction in the frequency of intradialytic hypotension after decreasing dialysate sodium [9, 18]. Therefore, individualization of dialysate sodium mainly influences the IDWG and leads to better BP control in patients with poorly controlled BP and this group of patients is generally asymptomatic. On the other hand, this is not the case with hemodiynamically stable patients or hypotensive-prone patients, where individualization of dialysate sodium has no influence on BP. The sodium modeling in our patients, too, did not result in better BP control (patients had the usual drop in BP) and led to increase in IDWG requiring higher UF rate which consequently, favors occurrence of hypotension. The results of some studies also suggest that the sodium profiling method does not prevent the increase in interdialytic weight gain and thirst often seen with other forms of high-sodium dialysis, and probably does not reduce the incidence of side effects [10, 19].

Analysis of the subjective feeling of thirst showed only a significant decrease in the thirst score in the normotensive group of patients, with no influence in the hypertensive and hypotensive group. Our speculation is that this is a result of the good nutrition status of patients, accompanied by sufficient intake of fats and calories as well as water, and we also agree with Lindey who postulated that patients drink fluids due to non – salt related reasons, such as comfort, social drinking or personal convictions [20, 21].

The study had few limitations: BP was not measured in the interdialytic period and sodium balance was not assessed during dialysis. Furthermore, the number of patients in the compared groups (normo-, hyper- and hypotensive) was not consistent and balanced, since all the patients included in the study belonged to one dialysis center and it was not possible to increase their number. These results impose the need for additional study including more patients from multiple dialysis centers.

In conclusion, the optimal dialysate sodium is not well defined and it dependson clinical circumstances. In hypertensive and stable normotensive patients isonatremic dialysis, or dialysis with lower dialysate sodium should be performed. Higher dialysate sodium in stable patients and sodium modeling in hipotensive-prone patients increases IDWG, but has no influence on blood pressure, suggesting that some other factors are involved that require further investigations. In these groups of patients we suggest the ultrafiltrate sodium concentration to be used as a dialysate-sodium prescription for future investigations.
